# Knowledge and Attitude of Sudanese Emergency Registrars Towards the Use of Tissue Plasminogen Activator in the Management of Acute Ischemic Stroke

**DOI:** 10.7759/cureus.29908

**Published:** 2022-10-04

**Authors:** Hussein J Ahmed, Rawan Yassin, Dalia Yassin, Ibrahim Elkhidir

**Affiliations:** 1 Faculty of Medicine, University of Khartoum, Khartoum, SDN; 2 Emergency Medicine, Faculty of Medicine, Alzaiem Alazhari University, Khartoum, SDN

**Keywords:** emergency medicine registrars, tpa, attitude, knowledge, acute ischemic stroke

## Abstract

Background: Recent guidelines report that the administration of tissue plasminogen activator (tPA) within 4.5 hours enhances the clinical outcome of ischemic stroke. We assessed the knowledge and attitude of Sudanese emergency medicine registrars towards the use of tissue plasminogen activators in the management of acute ischemic stroke (AIS).

Methods: This is a descriptive, cross-sectional, hospital-based study. The study was conducted in emergency departments at Khartoum State Hospitals in Sudan during the period from May to July 2021. The study sample was 150 emergency medicine registrars who fulfilled the inclusion criteria of the study. Data was collected by using a self-administered questionnaire as a Google form that was sent to the study participants by email.

Results: Knowledge about tPA in the management of AIS at the emergency department was poor, average, and good in 54 (36%), 55 (36.7%), and 41 (27.3%) individuals, respectively. However, there was no significant difference in overall knowledge based on age; nevertheless, good and average knowledge levels were considerably higher among females, level 3 and level 4 of training, and years of experience 5-10 years (p-value = 0.05). The overall attitude of the participants was positive 62 (41.3%), neutral 45 (30%), and negative 43 (28.7%). The positive attitude regarding tPAs for patients with AIS was significantly associated with age 30-40 years, females, levels 3 and 4 of training, and experience 5-10 years (p-value < 0.05).

Conclusion: The overall knowledge and attitude of emergency medicine registrars were average to good, with a significant proportion of participants reporting low knowledge. The issues encountered by participants in the use of tPAs were the absence of a stroke team in hospitals, the absence of a protocol for the care pathway of AIS management in hospitals, and the absence of tPAs.

## Introduction

Cerebrovascular stroke (CVS) is considered the second leading cause of death and dementia [1]. Acute ischemic stroke (AIS) is the sudden onset of focal neurological deficit resulting from occlusion in the vascular supply of an area in the brain. The causes of AIS are thrombosis, embolism, and hypoperfusion as they can occlude cerebral blood vessels compromising blood supply to that area [2].

There are two areas during vessel occlusion; a core area where direct instant ischemia causes severe and irreversible brain damage, and an area of “penumbra” where ischemia-induced damage to the brain is mild to moderate, gradually increasing, and reversible [3].

AIS is a potentially treatable condition, and treatment aims to restore blood flow to the penumbra before the damage becomes irreversible [4,5]. When applied to appropriate patients within four to five hours from the onset of stroke, treatments such as intravenous thrombolysis have been shown to improve outcomes [6,7].

As AIS is an emergency condition, emergency physicians and emergency doctor registrars have to deal with it rapidly and efficiently. An effort should be made to detect patients suitable for thrombolysis then perform brain imaging and refer those patients to stroke teams, all within the appropriate time window, as the speed at which AIS is treated is directly related to the outcome [8,9].

Most major general hospitals could provide infrastructure for the tissue plasminogen activator (tPA) therapy, but physicians’ insufficient knowledge may contribute to the lesser use of such a valuable therapy. Many physicians would overemphasize certain adverse effects associated with this therapy such as intracranial hemorrhage [8]. As a result, physicians’ attitudes would have a negative impact on the patients or their families so much so that they will likely refuse the tPA therapy following consultation [10].

It is well established that recombinant tPA is beneficial in the treatment of acute ischemic stroke within four to five hours after the onset of symptoms [11]. A small percentage of stroke patients worldwide receive this medication, for example only 3% to 5% of stroke victims in the United States receive thrombolysis. A number of factors contribute to this underutilization, including a lack of public awareness about the identification and proper treatment of acute stroke symptoms and signs, the complexity of the stroke system of care, and the slow adoption of such practice in the medical community [12,13].

In the stroke system of care, emergency physicians and registrars play central roles; emergency medical services (EMS) participate in prehospital stroke care.

Arrival at stroke centers within the appropriate time frame is affected by on-scene recognition, pre-notification, bypass, and decisions regarding the direct transport of stroke patients [14,15]. It is the emergency physicians' responsibility to identify stroke patients eligible for thrombolysis, perform fast triage and imaging, and refer them to the stroke team in a timely manner [16]. If there is a lack of stroke expertise on-site, emergency physicians might administer tPA, assisted by remote neurologists.

Prior studies suggest emergency physicians are resistant to the use of tPA in stroke [17,18]. A 2005 survey of members of the American College of Emergency Physicians found that even in the ideal setting (defined as CT scanner availability, neuroradiology and neurology support, administrative support, appropriate candidate, etc), 40% of respondents were either “unlikely” or “uncertain” to use tPA [18]. If widespread emergency physician resistance to tPA use is confirmed, it represents a significant barrier to increasing acute stroke treatment in the community setting [19].

It is unknown whether the Sudanese emergency registrars are knowledgeable and engaged in the thrombolytic process in Sudan, despite many hospitals having begun thrombolytic programs. We aimed to assess and quantify the knowledge and attitude of Sudanese emergency physicians toward the use of tPA in acute ischemic stroke (AIS). This article was presented as an online conference presentation at the 2021 International Colloquium on Neurology and Brain Disorders on December 15, 2021.

## Materials and methods

Study design, area, and settings

This is a cross-sectional descriptive hospital-based study, conducted in Khartoum State Hospitals in Sudan during the period from May 2021- July 2021. We included all emergency registrars of the R1 (Rotation Year 1), R2, R3, and R4 batches who registered in the Sudan Medical Specialization Board. Those who refused to participate or who stopped their training or traveled abroad at the time of the study were excluded.

Sampling

We followed the total coverage sampling technique of all emergency registrars who fulfilled the inclusion criteria and accepted to participate in the study. Due to the unavailability of a few of the emergency registrars in the study area during the study period for several reasons including refusal to participate in the study, absence, and inability to access them online, the final sample size was 150 out of 230. The response rate was 65.2% representing two-thirds of the total population. 

Data collection tools and methods

The data was collected by using a self-administered questionnaire as a Google form that was sent to the study participants by email or other social media after explaining the aim of the study and taking their consent.

We divided the questionnaire into three sections. Demographic information and general characteristics are included in the first part of the questionnaire. Age, gender, country, year of rotation, years of experience, level of hospital designation for stroke, hospital type, number of stroke patients treated, stroke expertise availability, and clinical care pathways in the emergency department were all collected. The second part investigated knowledge, and the third part investigated attitudes concerning the use of tPA in AIS.

Data analysis

All statistical analysis was performed using IBM SPSS Statistics for Windows, Version 26.0 (Released 2019; IBM Corp., Armonk, New York, United States). Data were checked for completeness, coded, and entered into the SPSS. The knowledge part has 10 correct points; accordingly, the total knowledge of the participant was rated as follows: Poor < 3/10, Average 4/10 - 6/10, and Good 7/10 - 10/10. The attitude part had 6 points; accordingly, the total attitude of a participant was rated as follows: Negative < 2/6, Neutral 2/6-3/6, and Positive 4/6 - 6/6.

Ethical Considerations

Approval was taken from the Sudan Medical Specialization Board (SMSB) and the Educational Development Center (EDC). After informing the research participants fully about the nature of the study, informed consent was obtained before enrolling them in the study by their choosing the 'agree' option at the beginning of the questionnaire. No confidential information was collected.

## Results

**Figure 1:**
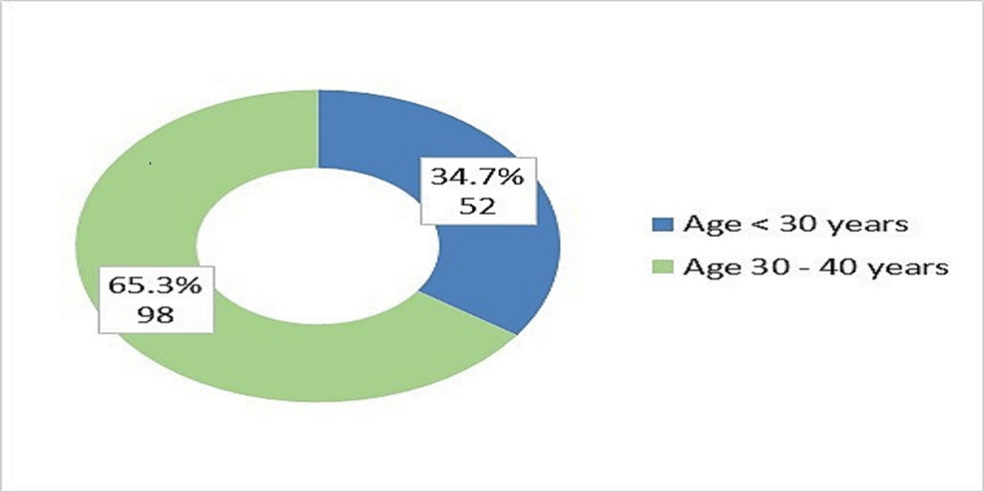
Distribution of the emergency medicine registrars according to age group

**Figure 2:**
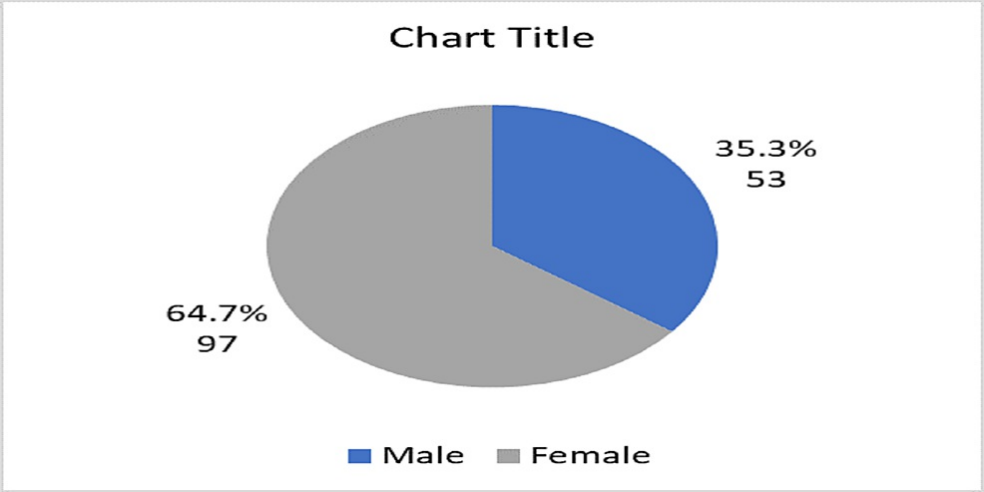
Distribution of the emergency medicine registrars according to gender

**Figure 3:**
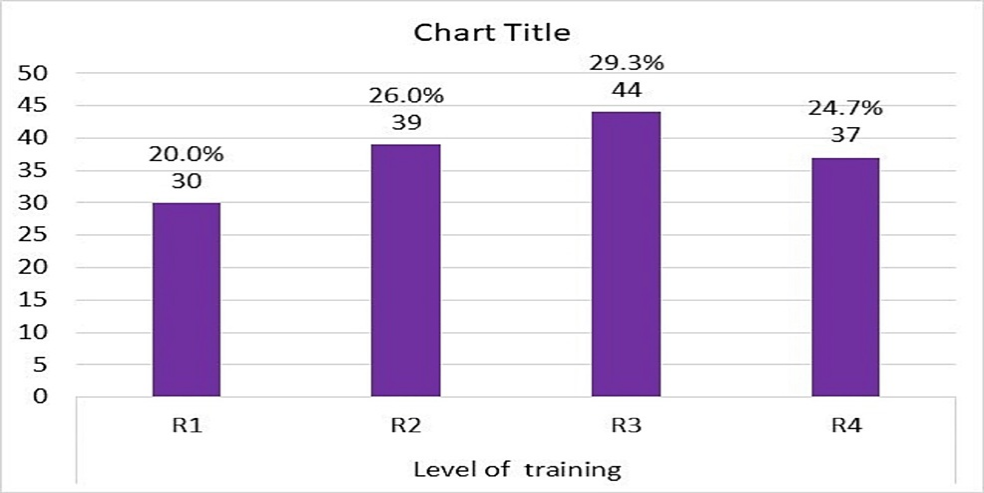
Distribution of the emergency medicine registrars according to the level of training

**Figure 4:**
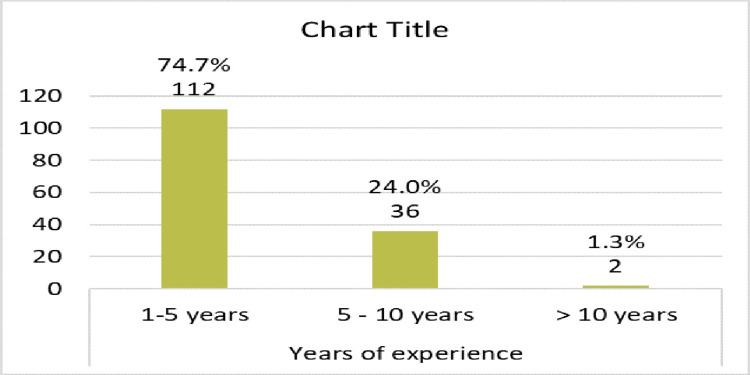
Distribution of the emergency medicine registrars according to years of experience

**Figure 5:**
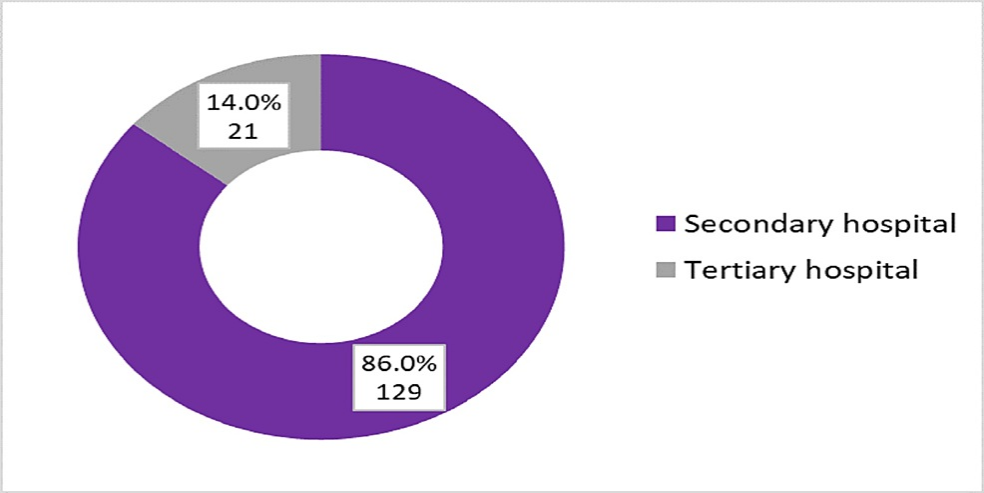
Distribution of the emergency medicine registrars according to the type of the hospital

About two-thirds of participants were females, and also two-thirds of participants were aged between 30 to 40 years old (Figure 1), and most of them were female (Figure 2). The training program consisted of four years of rotations (R1- R4) and the number of registrars in each year was almost similar (Figure 3). The majority of registrars had one to five years of experience (74%) (Figure 4), and the majority of them worked at secondary hospitals (Figure 5). They saw between one to five stroke patients per week.

**Figure 6:**
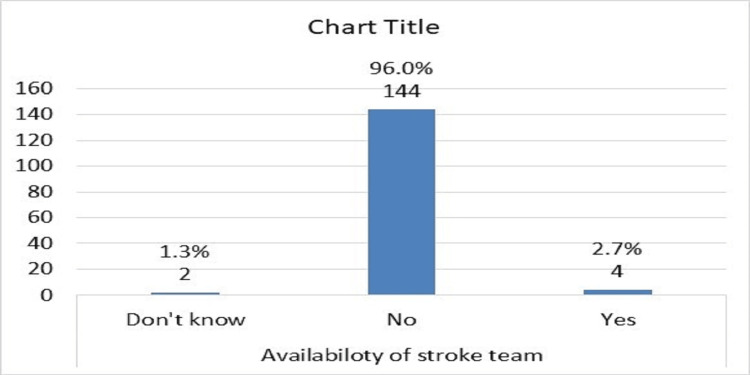
Distribution of the emergency medicine registrars according to availability of acute stroke team in the hospital

**Figure 7:**
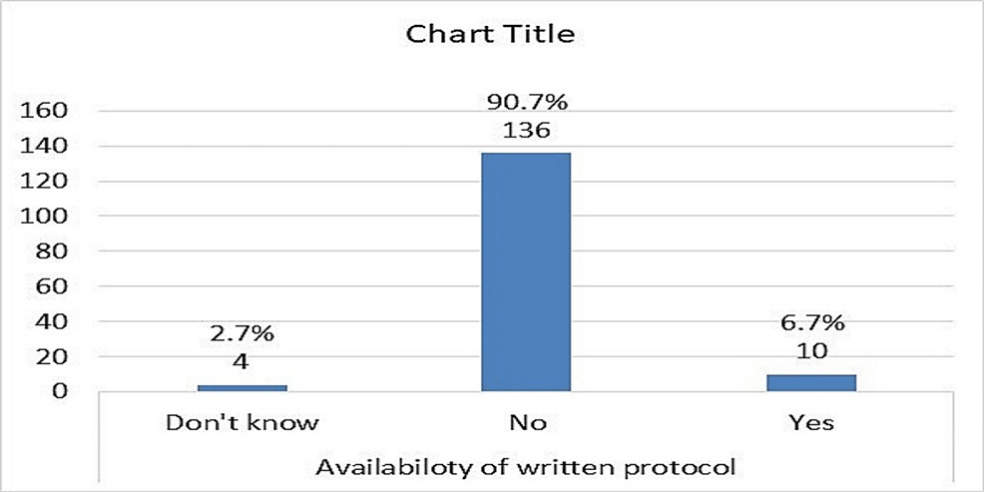
Distribution of the emergency medicine registrars according to availability of written protocol for care pathway of acute stroke management

There was no stroke team in 96% of hospitals (Figure 6) and there was no protocol care pathway in 90% of them (Figure 7). About 27 % of doctors had good knowledge about the use of tPA in stroke, 36% were average (moderate), and 36% had poor knowledge, and there was variation in knowledge regarding the items of inclusion and exclusion criteria of using tPA (Table 1; Table 2; Table 3; Table 4; and Table 5).

**Table 1:** Distribution of the emergency medicine registrars according to items of knowledge about the use of tPA tPA=tissue plasminogen activator

	N	%
Use of tissue plasminogen activators		
Yes	3	2.0
No	146	96.7
Don't know	1	1.3
Total	150	100.0
If no, the causes		
Not available	121	82.9
Delay time of patient presentation	12	8.2
No one knows about its use	13	8.9
Total	146	100.0
Self-rating of knowledge about tPA		
Well updated about the most recent literature and guidelines	15	10.0
General knowledge but acceptable	86	57.3
Poor knowledge	49	32.7
Total	150	100.0
tPA is an effective treatment		
Yes	132	88.0
No	3	2.0
Don't know	15	10.0
Total	150	100.0
Grade the level of evidence for the use of tPA		
Strong	57	38.0
Controversial	46	30.7
Week	11	7.3
Do not know	36	24.0
Total	150	100.0
Recommend tissue tPA for an eligible patient		
Yes	69	46.0
No	73	48.7
Uncertain	8	5.3
Total	150	100.0

**Table 2:** Distribution of the emergency medicine registrars according to reasons for not recommending tPA tPA=tissue plasminogen activator

If not recommended, the reasons	Yes		No		Total	
	N	%	N	%	N	%
Delay in patient arrival	51	69.9	22	30.1	73	100.0
Risk of hemorrhage	42	57.5	31	42.5	73	100.0
Cost	39	53.4	34	46.6	73	100.0
Lack of stroke expertise	28	38.4	45	61.6	73	100.0
Medico-legal inability	8	11.0	65	89.0	73	100.0
Lack of benefits	6	8.2	67	91.8	73	100.0

**Table 3:** Distribution of the emergency medicine registrars according to knowledge about criteria for use of tPA tPA=tissue plasminogen activator

Items	N	%
The window time to initiate thrombolysis in a patient with acute ischemic stroke		
0 - 3 hours	25	16.6
0 - 4.5 hours	105	70.0
0 - 6 hours	10	6.7
0 - 12 hours	10	6.7
Total	150	100.0
Control blood pressure and blood glucose before using tissue plasminogen activators		
Yes	132	88.0
No	7	4.7
Don't know	11	7.3
Total	150	100.0
Giving patient tissue plasminogen activators what is the standard dose		
0.9 mg/kg	37	28.0
0.1 mg/kg	26	19.7
0.5 mg/kg	69	52.3
Total	132	100.0
Know about inclusion and exclusion criteria for using tissue plasminogen activators		
Yes	44	29.3
No	106	70.7
Total	150	100.0

**Table 4:** Distribution of the emergency medicine registrars according to items of inclusion and exclusion criteria giving tPA NIH=National Institute of Health; tPA=tissue plasminogen activator

Inclusions	Yes		No		Total	
	N	%	N	%	N	%
Inclusion of stroke in less than 3 hours	13	29.5	31	70.5	44	100.0
Hemorrhagic stroke, arrival within the therapeutic window	12	27.3	32	72.7	44	100.0
Age equal to or more than 18	10	22.7	34	77.3	44	100.0
Stroke with neurological deficit and intracerebral hemorrhage	10	22.7	34	77.3	44	100.0
Brain tumor	10	22.7	34	77.3	44	100.0
Within the treatment window - recent intracerebral bleeding	10	22.7	34	77.3	44	100.0
Bleeding	8	18.2	36	81.8	44	100.0
Age less than 18 and more than 80 exclusion criterion	8	18.2	36	81.8	44	100.0
History of intracranial neoplasm	8	18.2	36	81.8	44	100.0
Internal bleeding	7	15.9	37	84.1	44	100.0
Time of symptom onset more than 4.5 hours	7	15.9	37	84.1	44	100.0
Current intracranial hemorrhage, within 4.5 hours	7	15.9	37	84.1	44	100.0
Inclusion age 18 years or above. Exclusion intracerebral bleeding	6	13.6	38	86.4	44	100.0
Recent major surgery	6	13.6	38	86.4	44	100.0
Age more than 69+NIH score less than 25	6	13.6	38	86.4	44	100.0
The patient arrives within 4.5h from the onset	5	11.4	39	88.6	44	100.0
Past history of a cerebral hemorrhage	5	11.4	39	88.6	44	100.0
Current use of anticoagulants	4	9.1	40	90.9	44	100.0

**Table 5:** Distribution of the emergency medicine registrars according to overall knowledge about the use of tPA for management of AIS at the emergency department tPA=tissue plasminogen activator; AIS=acute ischemic stroke

Total knowledge	N	%
Poor < 3/10	54	36.0
Average 4/10 - 6/10	55	36.7
Good 7/10 - 10/10	41	27.3
Total	150	100.0

The majority (41%) of doctors had a positive attitude toward the use of tPA, 30% had a neutral one, and 28% had a negative attitude (Table 6; Table 7). No significant difference in total knowledge according to age was found; however, a high percentage of good and average level of knowledge was associated with females, level 3 and level 4 of rotation years, and years of experience 5-10 years (p-value < 0.05) (Table 8), and the attitude of using tPA for AIS patients was significantly associated with age 30-40 years, females, level 3 and 4 of training, and experience 5-10 years (P-value < 0.05).

**Table 6:** Distribution of the emergency medicine registrars according to items of attitudes about the use of tPA for management of AIS at the emergency department tPA=tissue plasminogen activator

Items of attitudes	N	%
Feel confident about your ability to administer the use of tissue plasminogen activators		
Yes	68	45.3
No	82	54.7
Total	150	100.0
Support of hospitals' administration of tPA in acute ischemic stroke		
Yes	130	86.7
No	6	4.0
Uncertain	14	9.3
Total	150	100.0
tPA is the standard of care for ischemic stroke within a window in eligible patients		
Yes	118	78.7
No	10	6.7
Don't know	22	14.6
Total	150	100.0
In the absence of stroke expertise, what do you recommend		
No tissue plasminogen activators should be offered	21	14.0
Train emergency doctor to give tissue plasminogen activator	119	79.3
Train internists to give tissue plasminogen activators	2	1.3
Establish telestroke	8	5.4
Total	150	100.0
Willing to be enrolled in training to administer tissue plasminogen		
Yes	121	80.7
No	7	4.7
Uncertain	22	14.6
Total	150	100.0
Would you be willing to administer IV tPA using telestroke/ Remote consultation?		
Yes	120	80.0
No	7	4.7
Uncertain	23	15.3
Total	150	100.0

**Table 7:** Distribution of the emergency medicine registrars according to overall attitudes about the use of tPA for management of AIS at the emergency department tPA=tissue plasminogen activator

Attitudes	N	%
Negative < 2/6	43	28.7
Neutral 2/6-3/6	45	30.0
Positive 4/6 - 6/6	62	41.3
Total	150	100.0

**Table 8:** Distribution of the emergency medicine registrars according to overall knowledge about the use of tPA for management of AIS at the emergency department in relation to demographic features tPA=tissue plasminogen activator; AIS=acute ischemic stroke

		Total knowledge						
		Poor < 3/10		Average 4/10 - 6/10		Good 7/10 - 10/10		P value
		N	%	N	%	N	%	
Age	< 30 years	15	27.8	21	38.2	16	39.0	0.065
	30 - 40 years	39	72.2	34	61.8	25	61.0	
	Total	54	100.0	55	100.0	41	100.0	
Gender	Male	25	46.3	22	40.0	6	14.6	0.014
	Female	29	53.7	33	60.0	35	85.4	
	Total	54	100.0	55	100.0	41	100.0	
Position (level of training)	R1	21	38.9	9	16.4	0	0.0	0.022
	R2	18	33.3	17	30.9	4	9.8	
	R3	9	16.7	20	36.4	15	36.6	
	R4	6	11.1	9	16.4	22	53.7	
	Total	54	100.0	55	100.0	41	100.0	
Years of experience	1-5 years	54	100.0	46	83.6	12	29.3	0.018
	5 - 10 years	0	0.0	9	16.4	27	65.9	
	> 10 years	0	0.0	0	0.0	2	4.9	
	Total	54	100.0	55	100.0	41	100.0	

## Discussion

This study recruited 150 emergency medicine registrars to examine their knowledge and attitudes on the use of tPA for AIS patients in Khartoum State hospitals from May to July 2021.

Approximately, two-thirds of the participants, 98 (65.3%) were between the ages of 30 and 40, and 52 (34.7%) were under the age of 30. Females made up 97 (or 64.7%) of the participants, while males made up the remaining 53 (35.3%). This was in contrast to Alharbi et al. in Saudi Arabia who identified 81 respondents, (61.7%) were males and (38.3%) were females. The disparity could be attributed to a discrepancy in the distribution of male and female emergency department doctors between Sudan and Saudi Arabia [20].

Knowledge about tPA in the management of AIS at the emergency department was poor, average, and good in 54(36%), 55 (36.7%), and 41 (27.3%) individuals, respectively. However, there was no significant difference in overall knowledge based on age; nevertheless, good and average knowledge levels were considerably higher among females, level 3 and level 4 of training, and years of experience 5-10 years (P value 0.05); a similar result was reported by Brown et al. [17].

When asked if they were comfortable using tPA without first consulting with a senior, 65% said no. The evidence behind the use of tPA in stroke was compelling for 49% of those polled, while 30% were undecided. Non-emergency medicine board qualification, older age, and a smaller hospital practice environment were all related to positive opinions. El-khatib et al. studied Egyptian emergency physicians' awareness of tPA treatment use in AIS. The total knowledge of the study group was good, with a score of 7±1.8/11 out of 120 emergency physicians. Except for one question about blood glucose management prior to tPA use, the majority of participants (more than 50%) mentioned the correct answers. A significant positive correlation was noted between the knowledge score and age [21].

According to the study, 69 (46%) of respondents recommended tPA for eligible AIS patients, while 73 (48.7%) did not, and 8 (5.3%) were uncertain. The most prevalent reasons given by the 73 participants who do not recommend tPA were delay in patient arrival (51.9%), risk of bleeding (42.5%), cost (39.4%), lack of stroke expertise (28.4%), medico-legal (8.1%), and a lack of benefits 6 (8.2%). This was similar to the study by Al Khathaami et al., who assessed Saudi emergency physicians' knowledge and attitudes toward tPA usage within 4.5 hours of onset in acute ischemic stroke. According to their findings, half of the participants believe the evidence for tPA use in stroke within 4.5 hours of stroke onset is controversial, and 41% advise against its use due to a lack of proven efficacy (37%), the risk of hemorrhagic complications (35%), a lack of stroke expertise (21%), and medico-legal liability (9%) [22].

The overall attitude of the participants in this survey was positive 62 (41.3%), neutral 45 (30%), and negative 43% (28.7%). Positive attitudes about tPA for AIS patients were substantially associated with age 30-40 years, females, levels 3 and 4 of training, and experience 5-10 years (P value < 0.05).

The participants reported problems with the use of tPAs, including the absence of a stroke team in hospitals 144 (96%), the absence of protocols for the care pathway of AIS management in hospitals 136 (90.6%), and the absence of tissue plasminogen 8 (5.4%) [23]. Similar barriers were reported by Meur et al., as environmental and patient factors. Internal obstacles for the physician were a lack of familiarity with and motivation to follow the guidelines, as well as a lack of self-efficacy and outcome expectancy [24]. Baatiema et al. explored the opinions of stroke care providers on the barriers to providing optimal acute stroke care in Ghanaian hospital settings. There were four substantial hurdles and 12 barrier subthemes identified. Barriers include those with the patient (financial constraints, delays, sociocultural or religious practices, discharge against medical advice, stroke denial), health system (inadequate medical facilities, lack of stroke care protocol, limited staff numbers, inadequate staff development opportunities), health professionals (poor collaboration, limited knowledge of stroke care interventions), and broader national health policy (lack of political will) [25].

## Conclusions

A crucial role is played by emergency registrars in stroke care. As a result of their poor knowledge and negative attitude toward stroke thrombolysis, tPA might not be administered in a timely and effective fashion. Educating, engaging, and involving Sudanese emergency registrars in stroke care is imperative. Emergency registrars are frontline practitioners, and they could be policymakers and should be included in the planning and implementation of stroke care.
